# Crystal structure of (*E*)-2-(4-meth­oxy­styr­yl)-2,3-di­hydro-1*H*-perimidine aceto­nitrile monosolvate

**DOI:** 10.1107/S1600536814017000

**Published:** 2014-08-01

**Authors:** A. Manimekalai, N. Vijayalakshmi, S. Selvanayagam

**Affiliations:** a Department of Chemistry, Faculty of Science, Annamalai University, Annamalainagar 608 002, India; b Department of Physics, Kalasalingam University, Krishnankoil 626 126, India

**Keywords:** crystal structure, perimidine derivative, bifurcated hydrogen bonding

## Abstract

The title compound, C_20_H_18_N_2_O·CH_3_CN, a perimidine deriv­ative, crystallized as an aceto­nitrile monosolvate. The planes of the naphthalene ring system and the meth­oxy­phenyl ring are oriented almost perpendicular to one another, with a dihedral angle of 87.61 (6)°. The conformation about the C=C bond is *E*. The hexa­hydro­pyrimidine ring has an envelope conformation, with the methine C atom as the flap. In the crystal, the mol­ecules are linked by N—H⋯N hydrogen bonds involving the aceto­nitrile solvent mol­ecule as acceptor, forming zigzag chains propagating along [100].

## Related literature   

For the diverse range of biological activities of perimidines, see: Bu *et al.* (2001 ▶); Ivica *et al.* (2008 ▶); Azeez & Salih (2014 ▶). For a related structure, see: Maloney *et al.* (2013 ▶).
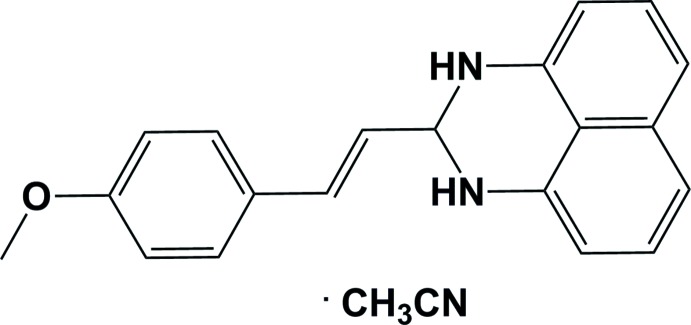


## Experimental   

### Crystal data  

C_20_H_18_N_2_O·C_2_H_3_N
*M* *_r_* = 343.42
Triclinic, 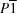

*a* = 7.8128 (7) Å
*b* = 8.4641 (7) Å
*c* = 14.7427 (14) Å
α = 79.513 (7)°
β = 83.861 (7)°
γ = 77.076 (7)°
*V* = 932.11 (15) Å^3^
*Z* = 2
Cu *K*α radiation
μ = 0.60 mm^−1^
*T* = 292 K
0.30 × 0.30 × 0.19 mm

### Data collection  

Oxford Diffraction Gemini/EOS CCD diffractometer
Absorption correction: multi-scan (*CrysAlis PRO*; Oxford Diffraction, 2008 ▶) *T*_min_ = 0.840, *T*_max_ = 0.890
5843 measured reflections
3569 independent reflections
2940 reflections with *I* > 2σ(*I*)
*R*_int_ = 0.023

### Refinement  

*R*[*F* ^2^ > 2σ(*F* ^2^)] = 0.048
*wR*(*F* ^2^) = 0.142
*S* = 1.03
3569 reflections
246 parameters
H atoms treated by a mixture of independent and constrained refinement
Δρ_max_ = 0.19 e Å^−3^
Δρ_min_ = −0.20 e Å^−3^

### 

Data collection: *CrysAlis PRO* (Oxford Diffraction, 2008 ▶); cell refinement: *CrysAlis PRO*; data reduction: *CrysAlis PRO*; program(s) used to solve structure: *SHELXS97* (Sheldrick, 2008 ▶); program(s) used to refine structure: *SHELXL2013* (Sheldrick, 2008 ▶); molecular graphics: *ORTEP-3 for Windows* (Farrugia, 2012 ▶) and *PLATON* (Spek, 2009 ▶); software used to prepare material for publication: *PLATON*.

## Supplementary Material

Crystal structure: contains datablock(s) I, global. DOI: 10.1107/S1600536814017000/su2762sup1.cif

Structure factors: contains datablock(s) I. DOI: 10.1107/S1600536814017000/su2762Isup2.hkl

Supporting information file. DOI: 10.1107/S1600536814017000/su2762Isup3.cml

**Fig. 1.**
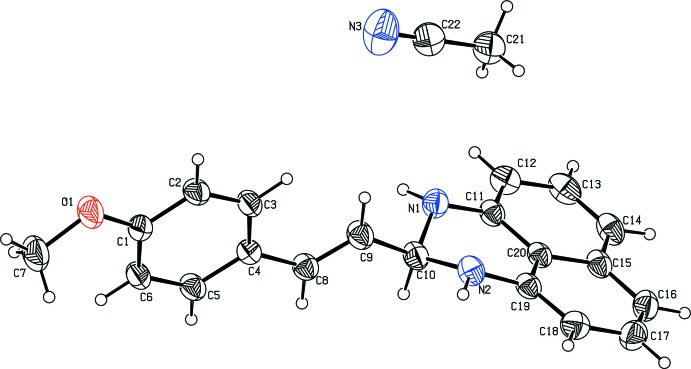
The molecular structure of the title compound, with atom labelling. Displacement ellipsoids are drawn at the 30% probability level.

. DOI: 10.1107/S1600536814017000/su2762fig1.tif
The mol­ecular structure of the title compound, with atom labelling. Displacement ellipsoids are drawn at the 30% probability level.

**Fig. 2.**
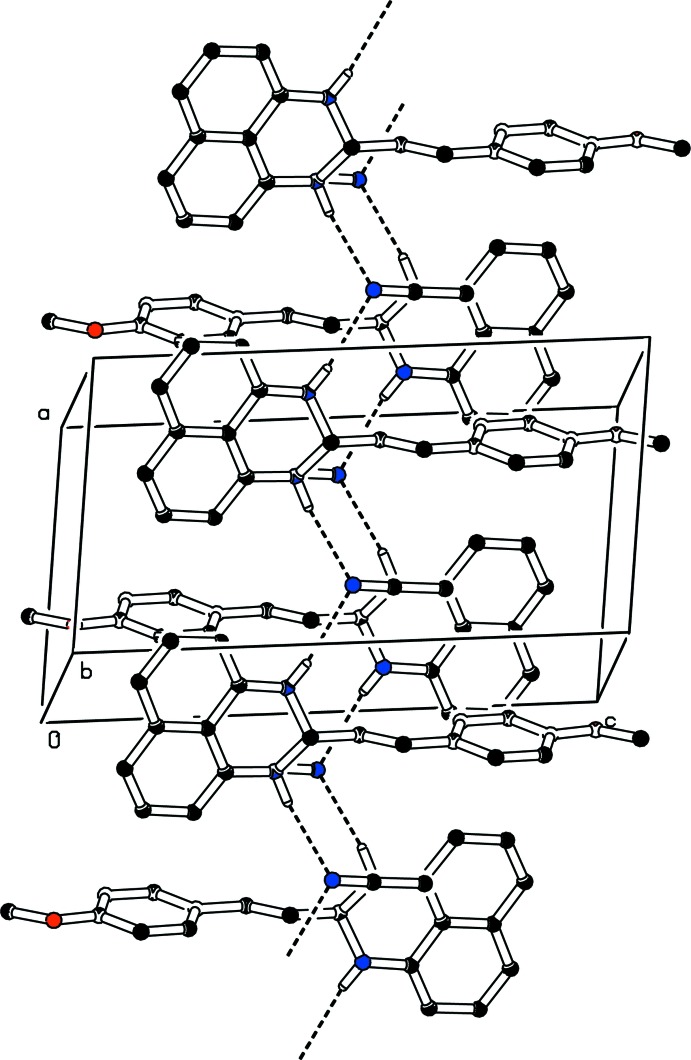
Crystal packing of the title compound, viewed along the b axis, showing the hydrogen bonds as dashed lines (see Table 1 for details; H atoms not involved in hydrogen bonding have been omitted for clarity).

. DOI: 10.1107/S1600536814017000/su2762fig2.tif
Crystal packing of the title compound, viewed along the b axis, showing the hydrogen bonds as dashed lines (see Table 1 for details; H atoms not involved in hydrogen bonding have been omitted for clarity).

CCDC reference: 1015577

Additional supporting information:  crystallographic information; 3D view; checkCIF report

## Figures and Tables

**Table 1:** Hydrogen-bond geometry (Å, °)

*D*—H⋯*A*	*D*—H	H⋯*A*	*D*⋯*A*	*D*—H⋯*A*
N1—H1⋯N3^i^	0.88 (2)	2.49 (2)	3.305 (3)	154.1 (15)
N2—H2⋯N3^ii^	0.84 (2)	2.44 (2)	3.234 (2)	156.8 (16)

Symmetry codes: (i) 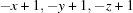; (ii) 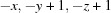.

## supplementary crystallographic information

### S1. Synthesis and crystallization

A mixture of 4-meth­oxy-trans-cinnamaldehyde (0.01 mol), 1,8-di­amino­naphthalene (0.01 mol) and a pinch of MgSO_4_.7H_2_O was finely grinded in a mortor. The mixture was then kept under microwave irradiation (LG Grill, Intellowave 160-800 W, consumption 800 W, output power 320 W and frequency 2450 MHz) operating in a cyclic mode to prevent intense boiling of the sample as well as aggregation of 320 W at 12 min. A brown colour solid separated out which was repeatedly recrystallized using CH_2_Cl_2_:CH_3_CN (1:5) to obtain brown crystals.

### S2. Refinement

Crystal data, data collection and structure refinement details are summarized in Table 2. Atoms H1 and H2 were located from a difference Fourier map and freely refined. The C-bound H atoms were positioned geometrically and allowed to ride on their parent atoms: C—H = 0.93-0.96 Å with U_iso_(H) = 1.5U_eq_(C-methyl) and U_iso_(H) = 1.2U_eq_ for other C atoms.

### Crystal data

**Table:**

C_20_H_18_N_2_O·C_2_H_3_N	*Z* = 2
*M**_r_* = 343.42	*F*(000) = 364
Triclinic, *P*1	*D*_x_ = 1.224 Mg m^−^^3^
*a* = 7.8128 (7) Å	Cu *K*α radiation, λ = 1.54184 Å
*b* = 8.4641 (7) Å	Cell parameters from 2420 reflections
*c* = 14.7427 (14) Å	θ = 3.8–26.7°
α = 79.513 (7)°	µ = 0.60 mm^−^^1^
β = 83.861 (7)°	*T* = 292 K
γ = 77.076 (7)°	Block, colourless
*V* = 932.11 (15) Å^3^	0.30 × 0.30 × 0.19 mm

### Data collection

**Table:**

Oxford Diffraction Gemini/EOS CCD diffractometer	2940 reflections with *I* > 2σ(*I*)
Radiation source: fine-focus sealed tube	*R*_int_ = 0.023
φ and ω scans	θ_max_ = 72.0°, θ_min_ = 3.1°
Absorption correction: multi-scan (*CrysAlis PRO*; Oxford Diffraction, 2008)	*h* = −8→9
*T*_min_ = 0.840, *T*_max_ = 0.890	*k* = −9→10
5843 measured reflections	*l* = −18→18
3569 independent reflections	

### Refinement

**Table:**

Refinement on *F*^2^	Secondary atom site location: difference Fourier map
Least-squares matrix: full	Hydrogen site location: mixed
*R*[*F*^2^ > 2σ(*F*^2^)] = 0.048	H atoms treated by a mixture of independent and constrained refinement
*wR*(*F*^2^) = 0.142	*w* = 1/[σ^2^(*F*_o_^2^) + (0.0768*P*)^2^ + 0.0999*P*] where *P* = (*F*_o_^2^ + 2*F*_c_^2^)/3
*S* = 1.03	(Δ/σ)_max_ < 0.001
3569 reflections	Δρ_max_ = 0.19 e Å^−^^3^
246 parameters	Δρ_min_ = −0.20 e Å^−^^3^
0 restraints	Extinction correction: *SHELXL*, Fc^*^=kFc[1+0.001xFc^2^λ^3^/sin(2θ)]^-1/4^
Primary atom site location: structure-invariant direct methods	Extinction coefficient: 0.038 (2)

### Special details

**Table:**

Geometry. All esds (except the esd in the dihedral angle between two l.s. planes) are estimated using the full covariance matrix. The cell esds are taken into account individually in the estimation of esds in distances, angles and torsion angles; correlations between esds in cell parameters are only used when they are defined by crystal symmetry. An approximate (isotropic) treatment of cell esds is used for estimating esds involving l.s. planes.

### Fractional atomic coordinates and isotropic or equivalent isotropic displacement parameters (Å^2^)

**Table:**

	*x*	*y*	*z*	*U*_iso_*/*U*_eq_	
O1	0.19042 (19)	0.58843 (14)	0.01925 (7)	0.0797 (4)	
N1	0.36331 (18)	0.27710 (16)	0.60754 (8)	0.0591 (3)	
H1	0.465 (2)	0.295 (2)	0.5801 (12)	0.070 (5)*	
N2	0.10070 (17)	0.19149 (16)	0.60104 (8)	0.0565 (3)	
H2	0.027 (2)	0.170 (2)	0.5698 (12)	0.066 (5)*	
N3	0.2463 (3)	0.7821 (3)	0.50695 (14)	0.1157 (7)	
C1	0.2221 (2)	0.49942 (17)	0.10443 (9)	0.0541 (3)	
C2	0.1430 (2)	0.57618 (17)	0.17864 (10)	0.0579 (4)	
H2A	0.0739	0.6816	0.1677	0.069*	
C3	0.16593 (19)	0.49800 (17)	0.26764 (9)	0.0534 (3)	
H3	0.1121	0.5512	0.3164	0.064*	
C4	0.26887 (17)	0.33940 (16)	0.28649 (9)	0.0469 (3)	
C5	0.34737 (19)	0.26561 (17)	0.21182 (9)	0.0550 (4)	
H5	0.4168	0.1603	0.2227	0.066*	
C6	0.3260 (2)	0.34347 (18)	0.12137 (10)	0.0577 (4)	
H6	0.3811	0.2912	0.0725	0.069*	
C7	0.2448 (3)	0.5081 (3)	−0.05893 (11)	0.0848 (6)	
H7A	0.3710	0.4773	−0.0645	0.127*	
H7B	0.2041	0.5813	−0.1137	0.127*	
H7C	0.1961	0.4116	−0.0514	0.127*	
C8	0.29403 (18)	0.25000 (17)	0.38074 (9)	0.0518 (3)	
H8	0.3531	0.1408	0.3858	0.062*	
C9	0.2431 (2)	0.30587 (18)	0.45874 (10)	0.0599 (4)	
H9	0.1876	0.4158	0.4560	0.072*	
C10	0.26927 (19)	0.20271 (18)	0.55165 (9)	0.0550 (4)	
H10	0.3362	0.0925	0.5446	0.066*	
C11	0.37511 (18)	0.20595 (16)	0.70001 (9)	0.0495 (3)	
C12	0.5038 (2)	0.22395 (19)	0.75213 (11)	0.0616 (4)	
H12	0.5903	0.2804	0.7247	0.074*	
C13	0.5045 (2)	0.1575 (2)	0.84619 (11)	0.0668 (4)	
H13	0.5919	0.1710	0.8805	0.080*	
C14	0.3811 (2)	0.0739 (2)	0.88851 (10)	0.0625 (4)	
H14	0.3848	0.0307	0.9511	0.075*	
C15	0.24639 (18)	0.05219 (16)	0.83788 (9)	0.0526 (3)	
C16	0.1127 (2)	−0.03118 (19)	0.87873 (11)	0.0644 (4)	
H16	0.1144	−0.0795	0.9407	0.077*	
C17	−0.0188 (2)	−0.0412 (2)	0.82776 (12)	0.0684 (4)	
H17	−0.1062	−0.0964	0.8555	0.082*	
C18	−0.02515 (19)	0.03028 (19)	0.73418 (11)	0.0608 (4)	
H18	−0.1172	0.0235	0.7009	0.073*	
C19	0.10345 (17)	0.10989 (16)	0.69160 (9)	0.0489 (3)	
C20	0.24349 (16)	0.12050 (15)	0.74293 (9)	0.0460 (3)	
C21	0.2512 (3)	0.6594 (2)	0.67886 (14)	0.0835 (5)	
H21A	0.3480	0.5668	0.6877	0.125*	
H21B	0.1430	0.6246	0.7000	0.125*	
H21C	0.2655	0.7410	0.7134	0.125*	
C22	0.2473 (2)	0.7278 (2)	0.58287 (14)	0.0749 (5)	

### Atomic displacement parameters (Å^2^)

**Table:**

	*U*^11^	*U*^22^	*U*^33^	*U*^12^	*U*^13^	*U*^23^
O1	0.1225 (10)	0.0607 (7)	0.0433 (6)	−0.0013 (6)	0.0027 (6)	−0.0027 (5)
N1	0.0629 (8)	0.0651 (8)	0.0494 (7)	−0.0174 (6)	−0.0017 (6)	−0.0063 (6)
N2	0.0556 (7)	0.0655 (8)	0.0475 (7)	−0.0064 (5)	−0.0112 (5)	−0.0100 (5)
N3	0.1015 (14)	0.168 (2)	0.0829 (13)	−0.0474 (14)	−0.0207 (10)	−0.0021 (13)
C1	0.0677 (8)	0.0491 (7)	0.0437 (7)	−0.0121 (6)	0.0009 (6)	−0.0056 (6)
C2	0.0710 (9)	0.0436 (7)	0.0528 (8)	0.0004 (6)	−0.0005 (7)	−0.0093 (6)
C3	0.0616 (8)	0.0503 (7)	0.0460 (7)	−0.0042 (6)	0.0024 (6)	−0.0147 (6)
C4	0.0472 (7)	0.0477 (7)	0.0460 (7)	−0.0083 (5)	−0.0015 (5)	−0.0112 (5)
C5	0.0619 (8)	0.0473 (7)	0.0508 (8)	0.0012 (6)	−0.0017 (6)	−0.0116 (6)
C6	0.0690 (9)	0.0543 (8)	0.0473 (7)	−0.0054 (6)	0.0059 (6)	−0.0168 (6)
C7	0.1177 (16)	0.0847 (12)	0.0441 (9)	−0.0088 (11)	0.0010 (9)	−0.0093 (8)
C8	0.0534 (7)	0.0503 (7)	0.0492 (7)	−0.0039 (6)	−0.0049 (6)	−0.0095 (6)
C9	0.0728 (9)	0.0531 (8)	0.0477 (8)	−0.0005 (7)	−0.0048 (7)	−0.0079 (6)
C10	0.0624 (8)	0.0534 (8)	0.0449 (7)	−0.0015 (6)	−0.0043 (6)	−0.0096 (6)
C11	0.0507 (7)	0.0481 (7)	0.0464 (7)	−0.0012 (5)	−0.0034 (5)	−0.0108 (5)
C12	0.0558 (8)	0.0629 (9)	0.0677 (9)	−0.0096 (7)	−0.0083 (7)	−0.0157 (7)
C13	0.0647 (9)	0.0715 (10)	0.0651 (9)	0.0021 (8)	−0.0226 (7)	−0.0230 (8)
C14	0.0692 (9)	0.0649 (9)	0.0459 (7)	0.0092 (7)	−0.0135 (7)	−0.0127 (6)
C15	0.0569 (8)	0.0471 (7)	0.0455 (7)	0.0080 (6)	−0.0027 (6)	−0.0097 (5)
C16	0.0738 (10)	0.0573 (8)	0.0506 (8)	−0.0005 (7)	0.0061 (7)	−0.0015 (6)
C17	0.0660 (9)	0.0624 (9)	0.0722 (10)	−0.0144 (7)	0.0123 (8)	−0.0081 (8)
C18	0.0531 (8)	0.0610 (9)	0.0688 (9)	−0.0100 (6)	−0.0021 (7)	−0.0154 (7)
C19	0.0499 (7)	0.0450 (7)	0.0481 (7)	0.0011 (5)	−0.0038 (5)	−0.0119 (5)
C20	0.0474 (7)	0.0419 (6)	0.0443 (7)	0.0033 (5)	−0.0029 (5)	−0.0111 (5)
C21	0.0940 (13)	0.0738 (11)	0.0794 (12)	−0.0137 (10)	0.0011 (10)	−0.0126 (9)
C22	0.0647 (10)	0.0877 (12)	0.0764 (12)	−0.0204 (9)	−0.0083 (8)	−0.0161 (10)

### Geometric parameters (Å, º)

**Table:**

O1—C1	1.3581 (16)	C9—C10	1.4917 (19)
O1—C7	1.422 (2)	C9—H9	0.9300
N1—C11	1.3902 (17)	C10—H10	0.9800
N1—C10	1.4630 (19)	C11—C12	1.379 (2)
N1—H1	0.882 (18)	C11—C20	1.4170 (19)
N2—C19	1.3869 (17)	C12—C13	1.398 (2)
N2—C10	1.4489 (19)	C12—H12	0.9300
N2—H2	0.842 (18)	C13—C14	1.355 (2)
N3—C22	1.130 (2)	C13—H13	0.9300
C1—C6	1.381 (2)	C14—C15	1.415 (2)
C1—C2	1.3913 (19)	C14—H14	0.9300
C2—C3	1.3685 (19)	C15—C16	1.412 (2)
C2—H2A	0.9300	C15—C20	1.4144 (18)
C3—C4	1.3983 (19)	C16—C17	1.362 (2)
C3—H3	0.9300	C16—H16	0.9300
C4—C5	1.3830 (18)	C17—C18	1.403 (2)
C4—C8	1.4666 (18)	C17—H17	0.9300
C5—C6	1.3852 (19)	C18—C19	1.368 (2)
C5—H5	0.9300	C18—H18	0.9300
C6—H6	0.9300	C19—C20	1.4226 (19)
C7—H7A	0.9600	C21—C22	1.429 (3)
C7—H7B	0.9600	C21—H21A	0.9600
C7—H7C	0.9600	C21—H21B	0.9600
C8—C9	1.312 (2)	C21—H21C	0.9600
C8—H8	0.9300		
			
C1—O1—C7	118.12 (13)	N2—C10—H10	109.7
C11—N1—C10	116.49 (12)	N1—C10—H10	109.7
C11—N1—H1	113.1 (11)	C9—C10—H10	109.7
C10—N1—H1	112.6 (11)	C12—C11—N1	122.29 (14)
C19—N2—C10	116.97 (11)	C12—C11—C20	119.28 (13)
C19—N2—H2	114.5 (12)	N1—C11—C20	118.32 (12)
C10—N2—H2	114.7 (12)	C11—C12—C13	120.18 (15)
O1—C1—C6	125.11 (13)	C11—C12—H12	119.9
O1—C1—C2	115.59 (13)	C13—C12—H12	119.9
C6—C1—C2	119.30 (13)	C14—C13—C12	121.58 (14)
C3—C2—C1	120.63 (13)	C14—C13—H13	119.2
C3—C2—H2A	119.7	C12—C13—H13	119.2
C1—C2—H2A	119.7	C13—C14—C15	120.31 (14)
C2—C3—C4	121.10 (12)	C13—C14—H14	119.8
C2—C3—H3	119.5	C15—C14—H14	119.8
C4—C3—H3	119.5	C16—C15—C20	118.76 (13)
C5—C4—C3	117.41 (12)	C16—C15—C14	122.73 (14)
C5—C4—C8	119.73 (12)	C20—C15—C14	118.49 (14)
C3—C4—C8	122.85 (12)	C17—C16—C15	120.21 (14)
C4—C5—C6	122.15 (12)	C17—C16—H16	119.9
C4—C5—H5	118.9	C15—C16—H16	119.9
C6—C5—H5	118.9	C16—C17—C18	121.29 (15)
C1—C6—C5	119.41 (13)	C16—C17—H17	119.4
C1—C6—H6	120.3	C18—C17—H17	119.4
C5—C6—H6	120.3	C19—C18—C17	120.33 (14)
O1—C7—H7A	109.5	C19—C18—H18	119.8
O1—C7—H7B	109.5	C17—C18—H18	119.8
H7A—C7—H7B	109.5	C18—C19—N2	123.57 (13)
O1—C7—H7C	109.5	C18—C19—C20	119.56 (13)
H7A—C7—H7C	109.5	N2—C19—C20	116.74 (12)
H7B—C7—H7C	109.5	C15—C20—C11	120.15 (12)
C9—C8—C4	127.69 (13)	C15—C20—C19	119.81 (13)
C9—C8—H8	116.2	C11—C20—C19	119.98 (12)
C4—C8—H8	116.2	C22—C21—H21A	109.5
C8—C9—C10	123.58 (14)	C22—C21—H21B	109.5
C8—C9—H9	118.2	H21A—C21—H21B	109.5
C10—C9—H9	118.2	C22—C21—H21C	109.5
N2—C10—N1	106.95 (11)	H21A—C21—H21C	109.5
N2—C10—C9	110.23 (12)	H21B—C21—H21C	109.5
N1—C10—C9	110.56 (12)	N3—C22—C21	179.1 (2)
			
C7—O1—C1—C6	10.1 (3)	C11—C12—C13—C14	0.2 (2)
C7—O1—C1—C2	−170.59 (16)	C12—C13—C14—C15	−0.2 (2)
O1—C1—C2—C3	−179.98 (14)	C13—C14—C15—C16	−178.84 (14)
C6—C1—C2—C3	−0.7 (2)	C13—C14—C15—C20	−0.5 (2)
C1—C2—C3—C4	−0.1 (2)	C20—C15—C16—C17	−1.8 (2)
C2—C3—C4—C5	0.5 (2)	C14—C15—C16—C17	176.61 (14)
C2—C3—C4—C8	−178.77 (13)	C15—C16—C17—C18	0.0 (2)
C3—C4—C5—C6	−0.2 (2)	C16—C17—C18—C19	1.0 (2)
C8—C4—C5—C6	179.08 (13)	C17—C18—C19—N2	−176.13 (13)
O1—C1—C6—C5	−179.82 (14)	C17—C18—C19—C20	−0.3 (2)
C2—C1—C6—C5	0.9 (2)	C10—N2—C19—C18	−152.05 (14)
C4—C5—C6—C1	−0.5 (2)	C10—N2—C19—C20	31.99 (17)
C5—C4—C8—C9	173.37 (16)	C16—C15—C20—C11	179.55 (12)
C3—C4—C8—C9	−7.4 (2)	C14—C15—C20—C11	1.10 (19)
C4—C8—C9—C10	177.61 (13)	C16—C15—C20—C19	2.48 (18)
C19—N2—C10—N1	−54.88 (16)	C14—C15—C20—C19	−175.97 (12)
C19—N2—C10—C9	−175.12 (11)	C12—C11—C20—C15	−1.08 (19)
C11—N1—C10—N2	51.33 (16)	N1—C11—C20—C15	−177.36 (11)
C11—N1—C10—C9	171.35 (12)	C12—C11—C20—C19	175.98 (12)
C8—C9—C10—N2	−116.90 (17)	N1—C11—C20—C19	−0.30 (19)
C8—C9—C10—N1	125.05 (16)	C18—C19—C20—C15	−1.48 (19)
C10—N1—C11—C12	157.87 (14)	N2—C19—C20—C15	174.65 (11)
C10—N1—C11—C20	−25.97 (18)	C18—C19—C20—C11	−178.55 (12)
N1—C11—C12—C13	176.53 (13)	N2—C19—C20—C11	−2.42 (18)
C20—C11—C12—C13	0.4 (2)		

### Hydrogen-bond geometry (Å, º)

**Table:**

*D*—H···*A*	*D*—H	H···*A*	*D*···*A*	*D*—H···*A*
N1—H1···N3^i^	0.88 (2)	2.49 (2)	3.305 (3)	154.1 (15)
N2—H2···N3^ii^	0.84 (2)	2.44 (2)	3.234 (2)	156.8 (16)

Symmetry codes: (i) −*x*+1, −*y*+1, −*z*+1; (ii) −*x*, −*y*+1, −*z*+1.

## References

[bb1] Azeez, H. J. & Salih, K. M. (2014). *Res. Pharm. Biotechnol.* 3, 1–6.

[bb2] Bu, X., Deady, W. L., Finlay, J. G., Bagwey, C. B. & Denny, A. W. (2001). *J. Med. Chem.* 44, 2004–2014. 10.1021/jm010041l 11384245

[bb3] Farrugia, L. J. (2012). *J. Appl. Cryst.* **45**, 849–854.

[bb4] Ivica, D., Mirta, R., Visnja, V., Sandra, P. K., Marijeta, K., Ivvo, D. & Marina, C. (2008). Bioorg. Med. Chem. 16, 5189–5198.

[bb5] Maloney, S., Slawin, A. M. Z. & Woollins, J. D. (2013). *Acta Cryst.* E**69**, o246. 10.1107/S1600536813000986 PMC3569779 23424525

[bb6] Oxford Diffraction (2008). *CrysAlis PRO* Oxford Diffraction Ltd, Yarnton, England.

[bb7] Sheldrick, G. M. (2008). *Acta Cryst.* A**64**, 112–122. 10.1107/S0108767307043930 18156677

[bb8] Spek, A. L. (2009). *Acta Cryst.* D**65**, 148–155. 10.1107/S090744490804362X PMC2631630 19171970

