# Supplementary figures and images for: Crystal structure of (E)-2-(4-meth­oxy­styr­yl)-2,3-di­hydro-1H-perimidine aceto­nitrile monosolvate
Source: Acta Crystallogr Sect E Struct Rep Online. 2014 Aug 1;70(Pt 9):o959. doi: 10.1107/S1600536814017000 (PMC4186146; doi:10.1107/S1600536814017000)

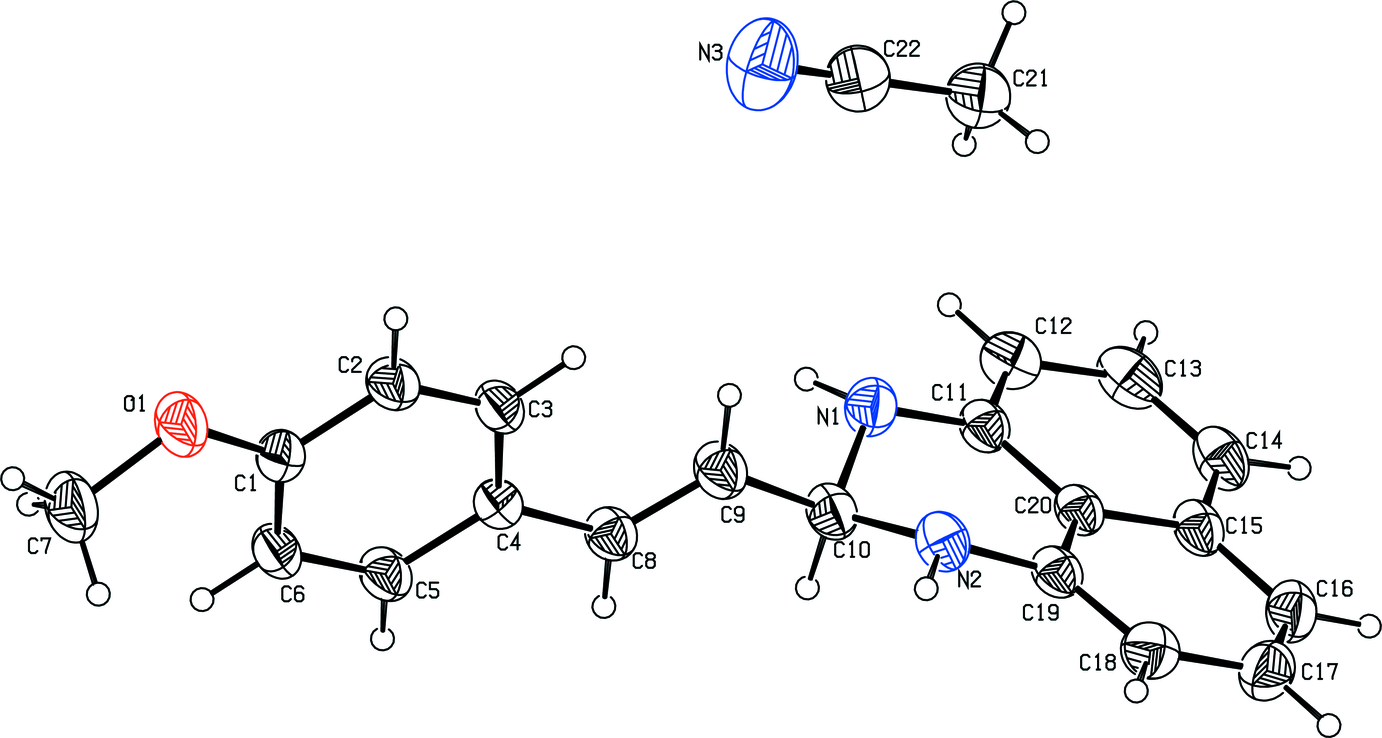

Supplement: Supplementary file 4 [file e-70-0o959-fig1.tif]

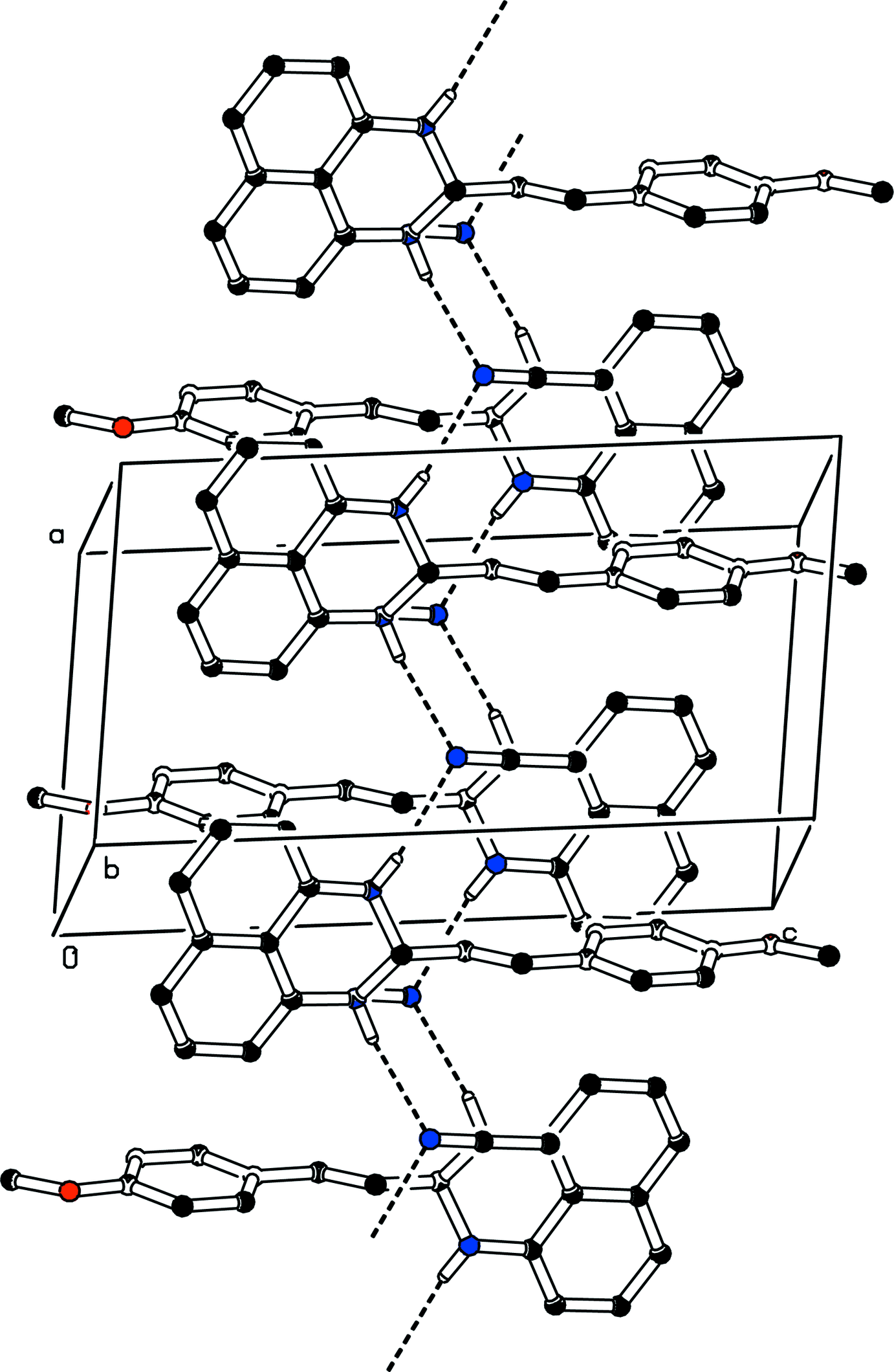

Supplement: Supplementary file 5 [file e-70-0o959-fig2.tif]
